# Apolipoprotein E in diet-induced obesity: a paradigm shift from conventional perception

**DOI:** 10.7555/JBR.32.20180007

**Published:** 2018-04-09

**Authors:** Kyriakos E. Kypreos, Eleni A. Karavia, Caterina Constantinou, Aikaterini Hatziri, Christina Kalogeropoulou, Eva Xepapadaki, Evangelia Zvintzou

**Affiliations:** Department of Pharmacology, University of Patras Medical School, Rio Achaias, TK 26500, Greece.; Department of Pharmacology, University of Patras Medical School, Rio Achaias, TK 26500, Greece.; Department of Pharmacology, University of Patras Medical School, Rio Achaias, TK 26500, Greece.; Department of Pharmacology, University of Patras Medical School, Rio Achaias, TK 26500, Greece.; Department of Pharmacology, University of Patras Medical School, Rio Achaias, TK 26500, Greece.; Department of Pharmacology, University of Patras Medical School, Rio Achaias, TK 26500, Greece.; Department of Pharmacology, University of Patras Medical School, Rio Achaias, TK 26500, Greece.

**Keywords:** apolipoprotein E, morbid obesity, white adipose tissue, energy metabolism

## Abstract

Apolipoprotein E (APOE) is a major protein component of peripheral and brain lipoprotein transport systems. APOE in peripheral circulation does not cross the blood brain barrier or blood cerebrospinal fluid barrier. As a result, peripheral APOE expression does not affect brain APOE levels and vice versa. Numerous epidemiological studies suggest a key role of peripherally expressed APOE in the development and progression of coronary heart disease while brain APOE has been associated with dementia and Alzheimer’s disease. More recent studies, mainly in experimental mice, suggested a link between Apoe and morbid obesity. According to the latest findings, expression of human apolipoprotein E3 (APOE3) isoform in the brain of mice is associated with a potent inhibition of visceral white adipose tissue (WAT) mitochondrial oxidative phosphorylation leading to significantly reduced substrate oxidation, increased fat accumulation and obesity. In contrast, hepatically expressed APOE3 is associated with a notable shift of substrate oxidation towards non-shivering thermogenesis in visceral WAT mitochondria, leading to resistance to obesity. These novel findings constitute a major paradigm shift from the widely accepted perception that APOE promotes obesity *via* receptor-mediated postprandial lipid delivery to WAT. Here, we provide a critical review of the latest facts on the role of APOE in morbid obesity.

## Adipose organ overview

Adipose organ is made of white adipose tissue (WAT) that is mainly responsible for lipid storage, and brown adipose tissue (BAT) that is mainly responsible for energy production (heat and ATP). Under certain circumstances, WAT may be activated metabolically and turn into BRITE (BRown Into whiTE) adipose tissue that is able to produce heat *via* non-shivering thermogenesis, but localizes in WAT depots^[1]^. Increased non-shivering thermogenesis is a result of elevated mitochondrial metabolic activity and mainly uncoupling protein 1 (Ucp1) function that mediates the metabolic conversion of free fatty acids to heat, thus contributing to the lean phenotype^[2^–^3]^. However, induction of WAT mitochondrial oxidative phosphorylation for ATP production, independently of Ucp1 increase, may also contribute to the lean phenotype^[4]^. The origin of newly formed brown adipocytes in adipose organ following exposure of animals to cold environment remains still under debate^[5^–^6]^, though some data indicate that they may be the result of transdifferentiation of white adipocytes into brown adipose tissue^[1^,^3^,^7]^. Metabolic activation of WAT into BRITE is considered a promising strategy for treatment of morbid obesity and numerous experimental drugs have been designed towards this goal, though to this date the molecular targets for such interventions remain vague.

## Apolipoprotein E (APOE) in brain and peripheral blood circulation

APOE is a major protein of the brain and peripheral lipoprotein transport systems. In humans, it is found in three natural isoforms, APOE2, APOE3 and APOE4 that differ at amino acid positions 112 and 158 where APOE2 contains two cysteine (cys) residues, APOE3 contains a cys and an arginine (arg) residue, and APOE4 contains two arg residues, respectively^[8]^. APOE is highly conserved among species. In mice, there is only one isoform of the protein. Sequence comparison indicates that mouse Apoe precursor is highly homologous to human APOE4 (71% identical, 82% positives, 3% sequence gaps). Though mouse Apoe is structurally similar to APOE4 due the presence of an arg residue at position 112, it contains threonine (thr) instead of arg at position 61. This amino acid substitution prevents the natural interaction between arg-61 and glutamic acid (glu)-255 in APOE4, making Apoe to function like APOE3^[9^–^10]^.

In humans, *Apoe* mRNA is primarily expressed in the liver, and to a lesser extent in the brain (pituitary gland, hypothalamus, cerebral cortex, hippocampus), kidney, lungs, spleen, colon, white adipose tissue, and other organs (https://www.proteinatlas.org/ENSG00000-130203-APOE/tissue).

Similar tissue distribution of *apoe* has been identified in mice^[11]^. *Apoe* gene regulation has been extensively studied in the liver and brain of mice, revealing distinct differences, characteristic of the site of expression^[11^–^12]^. Differences in gene expression have also been identified within different domains of the brain^[13^–^14]^. It should be noted that human and mouse *Apoe* promoters differ significantly, with less than 40% homology at 180 bp or more upstream of the two species transcription start site, suggesting that differences also exist in the regulation of *Apoe* expression in these two species^[15]^.

It is well-established that the brain is isolated from peripheral blood circulation and consequently from peripheral organs^[16]^ by i) the blood brain barrier (BBB) formed by cerebrovascular endothelial cells^[17]^, and ii) by the blood-cerebrospinal fluid barrier (BCSFB) composed by cuboidal epithelial cells of the choroid plexus^[18]^. Free cholesterol cannot cross either BBB or BCSFB^[19]^, thus all brain cholesterol originates from local *de novo* biosynthesis. However, it has been shown that some oxysterols that result from peripheral cholesterol oxidation can diffuse through BBB to both directions and possibly fulfill yet unexplored roles in cholesterol signaling and homeostasis^[20]^. Brain cholesterol synthesis is regulated by mechanisms like those observed in peripheral organs and tissues, with hydroxyl-methyl-glutaryl CoA reductase (HMGCoAR) activity defining the rate limiting step. Similarly, APOE in peripheral circulation does not cross either BBB or BCSFB^[21]^. Therefore, brain APOE is locally expressed by astrocytes and microglia^[22]^ in its different isoforms ApoE2, ApoE3, and ApoE4 with a frequency of 7%, 78%, and 15%, respectively^[23]^.

Interestingly, brain lipoproteins are also different from the lipoproteins present in the peripheral circulation^[16]^. Lipoproteins of human central nervous system (CNS) have been mainly studied in cerebrospinal fluid (CSF) that is readily accessible by noninvasive methods. It is particularly important to note that the APOE and APOB-containing lipoproteins of peripheral circulation, such as low-density lipoprotein (LDL), very low-density lipoprotein (VLDL), chylomicron and chylomicron remnants do not cross BBB or BCSFB and are undetectable in CSF^[21]^. In contrast, CSF lipoproteins have similar size and density to plasma HDL, thus are justifiably defined as "HDL-like particles"^[24]^. Four distinct lipoprotein subpopulations have been identified^[16]^. Notably, APOE and apolipoprotein A1 (APOA1) are the major protein constituents of CSF HDL-like particles^[24]^. Brain APOE has been strongly implicated in mechanisms associated with neurodegenerative diseases including Alzheimer’s disease^[25]^.

Peripherally expressed APOE is involved in plasma metabolism and clearance of triglyceride-rich lipoproteins (TRLs) from circulation^[26^–^27]^. Early work by Havel and coworkers established an inverse correlation between plasma APOE and TRLs levels^[28]^, while subsequent work showed that excess plasma APOE is an inhibitor of lipoprotein lipase (LPL). Plasma LPL is activated by Apolipoprotein C2 (APOC2) and inhibited Apolipoprotein C3 (APOC3)^[29]^. Excess APOE may inhibit LPL by displacing APOC2 from TRL particles^[30^–^31]^ or through direct protein-protein interaction where arginine residues in APOE are believed to play important role^[32]^.

APOE is also a structural component of HDL. It can promote the formation of HDL-like particles with the participation of lipid transporter ATP binding cassette A1 (ABCA1) and plasma enzyme Lecithin-cholesterol acyltransferase (LCAT) in a fashion similar to APOA1^[33^–^34]^. Numerous epidemiological studies suggested that APOE is associated with the development and progression of coronary heart disease in humans^[35^–^38]^, an observation that was further confirmed in experimental animals with the creation of the Apoe-deficient (*apoe*^*-/-*^) mouse^[39]^.

## Apolipoprotein E (APOE) in morbid obesity, a paradigm shift

More recent studies in mice strongly suggest a link between Apoe and morbid obesity. Work by Chiba *et al.*, showed that leptin and Apoe double deficient mice (*ob/ob x apoe*^*-/-*^) are resistant to body weight and adipose tissue gain when fed a high-fat/high-cholesterol diet, despite an increase in their plasma VLDL levels^[40]^. In another study, Huang *et al.* reported that *apoe*^*-/-*^ mice accumulate less body fat content and possess smaller adipocytes compared to wild type C57BL/6 controls^[41]^. Hofmann and coworkers extended further this observation by showing that *apoe*^*-/-*^ mice fed high-fat-high-sucrose diabetogenic diet for 24 weeks were resistant to diet-induced obesity and exhibited improved glucose uptake by muscle and brown adipose tissue^[42]^. The role of Apoe in obesity was also confirmed in the Ay^/+^ mice where deficiency in Apoe prevented the development of obesity, with decreased fat accumulation in the liver and adipose tissues^[43]^. Along the same line, mice expressing human APOE3 in place of mouse Apoe (*apoe3*^*knock-in*^) fed the standard western-type diet (21% fat) for 24 weeks were more sensitive to diet-induced obesity and related metabolic dysfunctions than wild-type C57BL/6 mice, while *apoe*^*-/-*^ mice were resistant to these conditions^[44]^. Work in humans confirmed the presence of elevated APOE levels in the plasma of morbidly obese subjects immediately prior to bariatric surgery while six months following the operation, APOE was barely detectable, establishing a correlation between plasma APOE levels and body mass index (BMI)^[45]^.

The important role of APOE in the clearance of TRLs from peripheral circulation led to a widely accepted perception among broader scientific community that APOE promotes obesity *via* receptor mediated postprandial lipid delivery to peripheral tissues, including BAT and WAT^[46]^. Indeed, lipoprotein-bound APOE is a natural ligand for the LDL-receptor (LDLR) and LDLR-related protein 1 (LRP1)^[47^–^49]^. APOE3 and APOE4 bind LDLR with a much higher affinity than APOE2^[50]^, while all three isoforms have similar binding affinities for LRP1^[51]^. However, LDLR has only a minor, if any, contribution to diet-induced obesity. Schreyer and coworkers reported that in contrast to *apoe*^*-/-*^ mice that remained lean, Ldlr-deficient (*ldlr*^*-/* -^) mice fed a diabetogenic high-fat high-sucrose diet (35.5% fat mainly from lard and 36.6% carbohydrate mainly from sucrose) became more obese than wild-type mice and developed severe hypertriglyceridemia and hyperleptinemia^[52]^. Along the same line, we also found that *ldlr*^*-/* -^ mice fed the standard Western-type diet became more obese than *apoe*^*-/-*^ mice and developed significant hepatic steatosis^[53]^.

Similarly, despite reports suggesting that adipose tissue Lrp1 may be implicated in diet-induced obesity^[54]^, more recent work showed that in the aP2-*cre* mouse, significant Cre activity is also found in ganglia of the peripheral nervous system (PNS), in adrenal medulla and in neurons throughout the CNS, proving that the aP2-*cre* mouse should no longer be used as a tool for adipose-tissue specific inactivation of genes^[55^–^56]^. Combining this information, along with our recent data showing that APOE promotes diet-induced obesity independent of its ability to mediate direct peripheral post-prandial lipid delivery to WAT^[57]^, an alternative interpretation of the data of Hofmann *et al*.^[54]^ is that the obesity resistant phenotype of floxed-*lrp1* mice crossed with aP2-*cre* mice is due to Lrp1 inactivation in PNS and CNS and not in adipose tissue, as originally suggested^[54]^. Confirming this alternative interpretation, tissue specific deletion of neuronal *lrp1* gene in brain resulted in an obese phenotype characterized by increased food intake, decreased energy consumption, and decreased leptin signaling^[58]^. This finding may further suggest that brain Lrp1 does not mediate the positive effects of brain Apoe on obesity development.

In an effort to investigate the mechanisms of APOE promoted diet-induced obesity, in a recent study, we employed four different mouse models: *Apoe*^*-/-*^, *Apoe3*^*brain*^, *Apoe3*^*knock-in*^, and *apoe*^*-/-*^ infected with an adenoassociated virus expressing APOE3 (AAV-E3)^[57]^. In the *Apoe3*^*brain*^ mouse, APOE3 expression is localized specifically in astrocytes and neuropil throughout development and into the adult period, with only trace amounts present in periphery^[14]^. On the other hand, *Apoe3*^*knock-in*^ mice express ubiquitously human APOE3 in place of mouse Apoe^[59]^, while *Apoe*^*-/-*^ mice infected with AAV-E3 express APOE3 in the liver which is the natural tropism of the virus^[60]^. We selected to study APOE3, which is the most common human APOE isoform^[8^,^27^,^61^–^65]^_._

**Fig.1:**
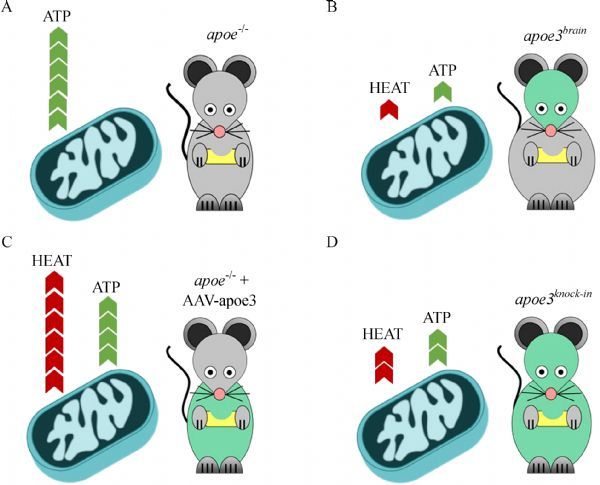
Effects of brain and peripheral APOE3 expression on visceral WAT mitochondrial metabolic activation. APOE3 expression affects visceral WAT mitochondrial metabolic activation in a fashion that is highly dependent on its site of expression and independent of postprandial dietary lipid deposition. A: Apoe-deficiency is associated with increased WAT mitochondrial oxidative phosphorylation towards ATP production leading to a lean phenotype; B: brain APOE3 expression is associated with a potent inhibition of visceral WAT mitochondrial oxidative phosphorylation leading to significantly reduced substrate oxidation, increased fat accumulation and obesity; C: peripherally expressed APOE3 is associated with a notable shift of substrate oxidation towards non-shivering thermogenesis in visceral WAT mitochondria, leading to resistance to obesity and a lean phenotype; D: Ubiquitously expressed APOE3 (ie. both in brain and periphery) is associated with potent inhibition of visceral WAT mitochondrial oxidative phosphorylation and non-shivering thermogenesis, further suggesting that brain APOE3 has a dominant effect over peripherally expressed APOE3 on WAT mitochondrial metabolism. Green color in mice denotes the site of APOE3 expression.

Interestingly, peripheral post-prandial lipid deposition to WAT did not correlate with body weight gain and obesity. Moreover, APOE3 exhibited a bimodal relation with visceral WAT mitochondrial metabolic activation that was highly dependent on its site of expression (brain *vs*. periphery) (***Fig. 1***). Our data showed that brain APOE3 expression in *Apoe3*^*brain*^ mice led to a potent inhibition of visceral WAT mitochondrial oxidative phosphorylation and consequently significantly reduced substrate oxidation, increased fat accumulation and obesity. To the contrary, hepatically expressed APOE3 in *apoe*^*-/-*^ mice infected with AAV-E3 was associated with a notable shift of substrate oxidation towards non-shivering thermogenesis in visceral WAT mitochondria, associated with resistance to obesity. Of note, brain APOE3-mediated inhibition was dominant over peripheral APOE3-mediated stimulation of WAT mitochondrial metabolic activity. The site-specific effects of APOE3 on obesity were independent of food consumption since all mouse strains consumed similar amounts of calories per day.

In agreement with our results, a very elegant study employing conditional *apoe* deletion mouse models showed that deletion of WAT-expressed *apoe* had no effect on morbid obesity while deletion of hepatically-expressed *apoe* resulted in rather higher adipocity in mice^[66]^, confirming the protective role of hepatically expressed APOE3 in obesity that we identified in our recently published work^[57]^. Therefore, the increased peripheral APOE levels observed in bariatric subjects prior to surgery^[45]^ may not represent a cause of morbid obesity but rather a protective mechanism triggered in response to nutrient surplus, aiming at promoting excess substrate oxidation in WAT mitochondria for energy production *via* non-shivering thermogenesis.

## Future directions

These latest findings constitute a major “paradigm shift” from the existing perception that peripherally expressed APOE promotes obesity *via* receptor-mediated postprandial lipid delivery to WAT. Contrasting this outdated view, the current data show that increasing hepatically expressed APOE may exert a significant beneficial effect against WAT expansion and morbid obesity development (***Fig.1***).

**Fig.2:**
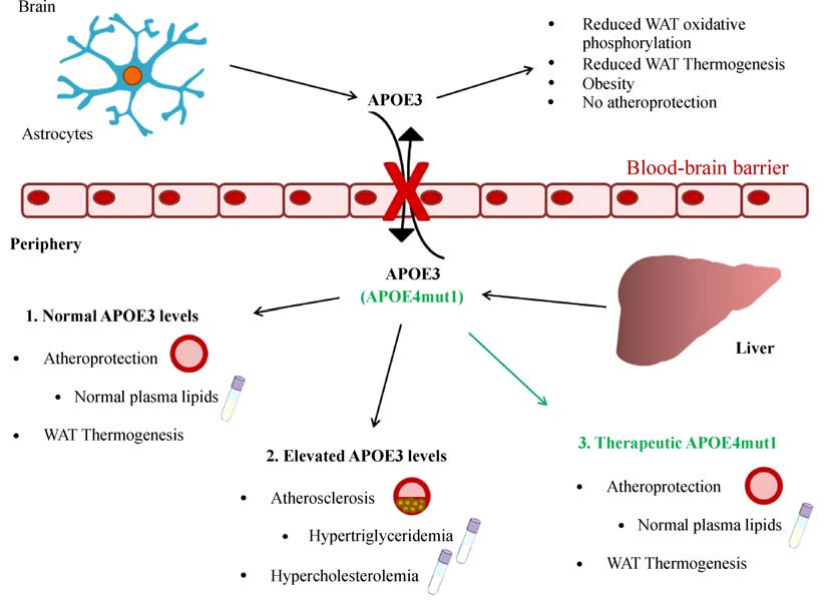
Working model summarizing the current understanding of APOE function. Effects of endogenously expressed APOE3 in the brain and in the periphery, in the context of atherosclerosis, dyslipidemia and morbid obesity. The potential benefit of therapeutic forms of APOE, such as APOE4mut1 [68], is also presented.

A major undesirable effect associated with increased peripheral wild-type (wt) APOE expression is the induction of severe hypertriglyceridemia and subsequently combined hyperlipidemia (***Fig.2***). Indeed, as detailed above plasma APOE levels show a direct correlation with plasma triglyceride levels^[28]^. Even the subtlest increase of plasma APOE concentration from physiological (2–5 mg/dL), triggers severe hypertriglyceridemia that is due to direct inhibition of plasma LPL activity^[32]^ and increased hepatic VLDL production^[67]^ (***Fig. 2***).

A solution to this limitation may be the use of recombinant APOE forms with improved biological functions^[47^,^68^–^69]^. We showed previously that APOE-induced hypertriglyceridemia requires the carboxyterminal domain 260-299 of APOE^[47]^, and specifically helix 8 that extends from aminoacids 260-270^[68]^. Using site directed mutagenesis. We created a recombinant APOE form termed APOE4mut1 (apoE4 [L261A, W264A, F265A, L268A, V269A]) that in mice corrects hypercholesterolemia without triggering hypertriglyceridemia even at concentration as high as 170 mg/dL^[68]^ (***Fig. 2***). Additional studies should determine if truncated or mutated APOE forms could also serve as biological anti-obesity drugs for WAT mitochondrial metabolic activation towards non-shivering thermogenesis^[47^,^68]^.

Despite these major advances in the understanding of the role of APOE in morbid obesity, the precise mechanisms underlying its opposing site-specific effects, including the effector molecules mediating the cross-talk between brain APOE and WAT mitochondrial metabolic activity, remain unclear. Importantly, the role of brain APOE-containing lipoproteins in body-weight gain needs further investigation.
